# Supplementary material for: Antisense regulation by transposon-derived RNAs in the hyperthermophilic archaeon Sulfolobus solfataricus
Source: EMBO Rep. 2013 Apr 12;14(6):527–33. doi: 10.1038/embor.2013.47 (PMC3674436; doi:10.1038/embor.2013.47)
Supplement: Supplementary Information [file embor201347s1.pdf]

Supplementary Information

Methods

Construction of Plasmids

Plasmid pMJ05-1183 was constructed as follows. The 3'UTR of ORF *1183* (bp 1075 to bp 1557) was amplified by PCR from *Sulfolobus solfataricus* (Sso) genomic DNA. The PCR product obtained with the forward primer 5'-CCGGGGGCCCCGGAATAACCCCAACCC-3', which contained an *ApaI* site (underlined) and the reverse primer 5'-GGCCCGGCCGCTAAGGTGCAATCATTTGC -3', which contained an *Eco52I* site (underlined), was cleaved and ligated into the corresponding sites of plasmid pSVA5 [1], resulting in plasmid pSVA5-1183. Plasmid pSVA5-1183 was then cleaved with *Eco52I* and *XmaJI* and the resulting fragment was ligated into the corresponding sites of plasmid pMJ05 [2], giving rise to plasmid pMJ05-1183.

For plasmid pMJ05-1183R26 the sequence 5'-CTTGGAATATCACGGATGTG-3' (bp 1097 to bp 1122 of Sso*1183*) was replaced with the sequence 5'-TCACATCCGTGATATTCGTATATGAG-3'. The *Sso*1183-3´UTR with the replaced sequence flanked by *ApaI* and *Eco52I* restriction sites was synthesized resulting in plasmid pCR2.1-1183R26 (Eurofins). Plasmid pCR2.1-1183R26 was cleaved with *ApaI* and *Eco52I*, and the fragment was ligated into the corresponding sites of plasmid pSVA5 [1], resulting in plasmid pSVA5-1183R26. Plasmid pSVA5-1183UTR26 was then cleaved with *Eco52I* and *XmaJI* and the resulting fragment was ligated into the corresponding sites of plasmid pMJ05, resulting in plasmid pMJ05-1183R26.

For plasmid pMJ05-Δ the sequence 5'-CCCAACCCACACCCTTGGAATATCACGGATGTGAGGAAAACCGCACATCTT-3' (bp 1084 to bp 1144 in *1183*) was replaced with sequence 5'-TTGGCTTTTGGTCAGAACGAATATCACCCTCTCAAATTCCAGGGCTGGTGTAA-3' resulting in a shorter transcript. For PCR mutagenesis the primers 5'-TTTGGGCCCCGGAATAATTGGCTTTTGGTCAGAACGAATATCACCCTCTCAAATTCCAGGGCTGGTGTAAGAGATAAATATTCAGTGAAGCATTCAATTG-3' and 5'-GGCCCGGCCGCCATGGAGGTGAAGAGGGCTAAGGTGCAATCATTTG-3' were used with chromosomal Sso DNA as template. The 482 bp PCR-product of the Sso *1183*-3´UTR with the replaced sequence was cleaved with *ApaI* and *Eco52I*, and ligated in the corresponding sites of plasmid pSVA5 [1], resulting in plasmid pSVA5-

Δ. Plasmid pSVA5-Δ was then cleaved with *Eco52I* and *XmaJI* and the fragment obtained was ligated into the corresponding sites of plasmid pMJ05, resulting in plasmid pMJ05-Δ.

For Plasmid pMJ05-6545-1183 an overlap PCR was performed using oligonucleotide 5'-CATGCCATGGATTTTTGGCAGAAACAGCGC-3' together with 5'-GGTTGGGGTTATTCCGCTAAGAAGTTTGTAGAGGAG-3' and oligonucleotide 5'-CTCCTCTACAAACTTCTTAGCGGAATAACCCCAACC-3' together with 5'-CGCGGATCCAGGTGAAGAGGGCTAAG-3' and genomic DNA as template. The second PCR was performed using the annealed PCR products as template with oligonucleotides 5'-CATGCCATGGATTTTTGGCAGAAACAGCGC-3' containing a *NcoI* site and 5'-CGCGGATCCAGGTGAAGAGGGCTAAG-3' containing a *BamH1* site. The PCR product was cleaved and ligated into the corresponding sites of plasmid pSVA5 [1], resulting in plasmid pSVA5-6454-1183. Plasmid pSVA5-Sso*6454*-1183 was then cleaved with *Eco52I* and *XmaJI* and the resulting fragment was ligated into the corresponding sites of plasmid pMJ05 [2], which gave rise to plasmid pMJ05-6545-1183.

Transformation of *Sso*

100–150 ng of the respective plasmid DNA was used for electroporation of exponentially grown *S. solfataricus* PH1-16 (Δ*pyrEF*; Δ*lacS*) as described in [2]. The cells were regenerated in medium containing uracil, and then selected in medium without uracil. After reaching an OD_{600} of 0.5, the cells were plated and single colonies were inoculated in liquid media without uracil. Genomic DNA was isolated and the presence of the respective plasmids was confirmed using the pMJ05 specific primers 5'-GGATGCTAAACAACTATTCAAACTG-3' and 5'-GTTGTGTGGAATTGTGAGCGGATAA-3'.

Phosphate limiting conditions

The strains P2, PH1-16(pMJ05), PH1-16(pMJ05-1183), PH1-16(pMJ05-1183R26), PH1-16(pMJ05-Δ), PH1-16(pMJ05-6545-1183) were grown in Brock's medium to an OD_{600} of 0.6, composed of Brock's salts (final concentration of 280 mg / l KH_2PO_4) supplemented with 0.2 % NZamine and 0.2 % glucose or arabinose. The cells were pelleted and washed before dilution to OD_{600} of 0.1 into Brock's medium without addition of KH_2PO_4 in 200 x Brock medium. After the cells reached an OD_{600} of 0.5,

the cells were diluted again in Brock's medium without KH_2PO_4 to an OD_{600} of 0.1. The cells were harvested when they reached an OD_{600} of 0.6 followed by extraction of total RNA (see below).

RT-PCR

Total RNA was isolated as described [3] and DNase I treated. cDNA was synthesized from 1 µg of total RNA using random hexamer oligos (Fermentas) and SuperScriptIII Reverse Transcriptase (Invitrogen). For RT-PCR, 10 ng or 500 ng cDNA from strains PH1-16(pMJ05), PH1-16(pMJ05-1183), PH1-16(pMJ05-1183R26), PH1-16(pMJ05-Δ), PH1-16(pMJ05-6545-1183) grown in either full medium (+P) or under phosphate limiting conditions (-P) was used as template in a PCR reaction with 30 cycles using GoTAQ Mastermix (Promega), and either the *lacS* (Sso *3019*) specific oligonucleotides (5'-ATGTACTCATTTCCAAATAGC-3' and 5´-ATCTCTACCGCTTCCATATCTTTATCCGT-3´), the *6454-1183* specific primers (5'-GATACAAAGTGATGGTAGTGG-3' and 5'-GTGGGTTTGTATCTCGAAAG-3') and the RNA-257_1 specific oligonucleotides (5'-GTGGGTTCGCACTAGACCTC-3' and 5'-CTTAGTGGGTTTGGGCTTCA-3'). As internal / loading controls the abundance of Sso *0412* mRNA, encoding the aIF2 gamma subunit, and that of 5S ribosomal RNA was determined. The Sso *0412* specific primers (5'-AGCCCAAGTTTCTGAGACGA-3' and 5'- TGCGATAACGCTTCCTCTTT-3'), and the 5S rRNA specific oligonucleotides 5'-CCACCCGGTCACAGTG-3' and 5'-CCCATCCCAGCTTAGTG-3' were used. The PCR products were separated on 6% polyacrylamide gels and the signals were quantified using Image J software.

qPCR

Total RNA was isolated as described [3] and DNase I treated. cDNA was synthesized from 1 µg of total RNA using random hexamer oligos (Fermentas) and SuperScriptIII Reverse Transcriptase (Invitrogen). For qPCR 10 ng cDNA from wildtype strain P2 and strains PH1-16(pMJ05), PH1-16(pMJ05-1183), PH1-16(pMJ05-1183R26), PH1-16(pMJ05-Δ), grown in either full medium (+P) or under phosphate limiting conditions (-P) was used as template. The cDNA was RNase H treated and qPCR analysis was performed with RNA-257_1 specific primers (5'-GTGGGTTCGCACTAGACCTC-3' and 5'-CTTAGTGGGTTTGGGCTTCA-3'), Sso *3019* (*lacS*) specific primers (5'-GGAAGCGGTAGAGATGGCTG-3' and 5'-CATCCGTGACCGTAACCTCC-3') and the Sso

1183 specific primers (5'-TGATGACGTTTGGAGCGTTA-3' and 5'-CAAAGCCCCAGAATAGTCCA-3'). The primers were designed with Primer 3 software (http://frodo.wi.mit.edu/primer3). As internal standard for the cDNA amount Sso *0412* specific primers (5'-AGCCCAAGTTTCTGAGACGA-3' and 5'-TGCGATAACGCTTCCTCTTT-3') were used. The real-time PCR mixture containing 2 x iQ SYBR green Supermix (Biorad) or 5x HOT FIREPol EvaGreen® qPCR Mix (Medibena), 10 ng cDNA and 250 nM of each primer was placed in a Real-time PCR cycler (Eppendorf Mastercycler) and the reaction was started at 95°C for 3 min or 15 min, followed by 40 cycles of 25s at 95°C, 20s at 58°C and 20s at 72°C. All reactions including the DNA standards and the negative control (no template) were done in triplicate. The fluorescence was measured at the last step of each cycle. After 40 cycles, a melting curve analysis was performed by raising the temperature from 45°C to 95°C every 15s, and by measuring the fluorescence at each cycle. The quantification was made using the software realplex 2.2 from a standard curve generated by titration of 10^1-10^6 copies of linearized plasmid pSAV5 harboring either the full length RNA-257_1 gene, Sso *3019*, Sso *1183* or Sso *0412*.

In vitro RNA transcription

The PCR-templates for synthesis of *1183* mRNA and the *1183* 3´UTR were prepared by using genomic *Sso* DNA as template together with the oligonucleotides 5'-CGGGATCCTAATACGACTCACTATAGGATGAATGTAAAGAACGTATTC-3' (*1183* mRNA) and 5'-CGGGATCCTAATACGACTCACTATAGGCCAGGTGGACTTATAG CTCTCAC-3' (*1183* 3´UTR) containing a T7 Promotor (underlined) and the reverse primer 5'- CGGAATTCGGATGCTAATGACTCTCTTTCG-3'. For RNA-257_1 primer 5'-CGGGATCCTAATACGACTCACTATAGGGTGAAGTGAACACACACCTC-3' and primer 5'-CGGAATTCCTTACTTAGTGGGTTTGGGC-3' were used. For transcription of the Sso *3148* 3´UTR, the primers 5'-TAATACGACTCACTATAGGATGAACTCCATACTGATGACG and 5'-GAACTCCATACTGATGACGG-3' were used. For transcription of the *lacS'*-3´UTR-*1183* we used plasmid pMJ05-1183 as template together with the oligonucleotides 5'-TAATACGACTCACTATAGGGTGAGATCACCAGAGGAAACG-3' (complementary to nts 887-907 of *lacS*) containing a T7 Promotor (underlined) and 5'-GGCCCGGCCGCCATGGAGGTGAAGAGGGCTAAGGTGCAATCATTTG-3'

(complementary to the 1183 3'UTR) resulting in a 5´ deletion of *lacS* mRNA (*lacS*') containing 584 nts of the coding region fused to the 1183 3'UTR.

Preparation of S30 Extracts

200 ml of a Sso culture (OD_{600} = 0.4) was harvested, washed and resuspended in buffer A (20 mM Tris / HCl pH 7.0; 10 mM Mg-acetate; 40 mM NH_4Cl; 1 mM DTT). The cells were lysed by snap freezing in liquid nitrogen followed by thawing at 37°C using 4 repetitions. The lysate was centrifuged at 30.000 x g for 30 min at 4°C. The S30 extract was adjusted to an OD_{280} of 30 µg / µl with buffer A and supplemented with glycerol to a final concentration of 10 %.

β-galactosidase assay

At an OD_{600} of 0.6, 2 ml cultures of strains PH1-16 (pMJ05), PH1-16 (pMJ05-1183), PH1-16(pMJ05-1183R26) and PH1-16 (pMJ05-Δ) growing either in Brock's medium or under phosphate limiting conditions (see above) were harvested and the cell-pellets were resuspended in 50 µl 10 mM Tris / HCl pH 8.0. Crude cell extracts were prepared by placing the cells 4 times in liquid nitrogen and then for 5 min at 37 °C. The extract was centrifuged for 30 min at 10.000 x g. To 50 µl of the supernatant 950 µl of pre-heated reaction buffer (50 mM sodiumphosphate pH 6.5, 4 mg / ml ONPG) were added and the mixture was incubated for 30 min at 70°C. After incubation, the OD_{420} was measured and the Miller units were calculated following the standard protocol.

REFERENCES

1. Albers, S.V., Jonuscheit, M., Dinkelaker, S., Urich, T., Kletzin, A., Tampe, R., Driessen, A.J., and Schleper, C. (2006). Production of recombinant and tagged proteins in the hyperthermophilic archaeon *Sulfolobus solfataricus*. *Appl Environ Microbiol* **72**, 102-111.

2. Jonuscheit, M., Martusewitsch, E., Stedman, K.M., and Schleper, C. (2003). A reporter gene system for the hyperthermophilic archaeon *Sulfolobus solfataricus* based on a selectable and integrative shuttle vector. *Mol Microbiol* **48**, 1241-1252.

3. Chomczynski, P., and Sacchi, N. (1987). Single-step method of RNA isolation by acid guanidinium thiocyanate-phenol-chloroform extraction. *Anal Biochem* **162**, 156-159.

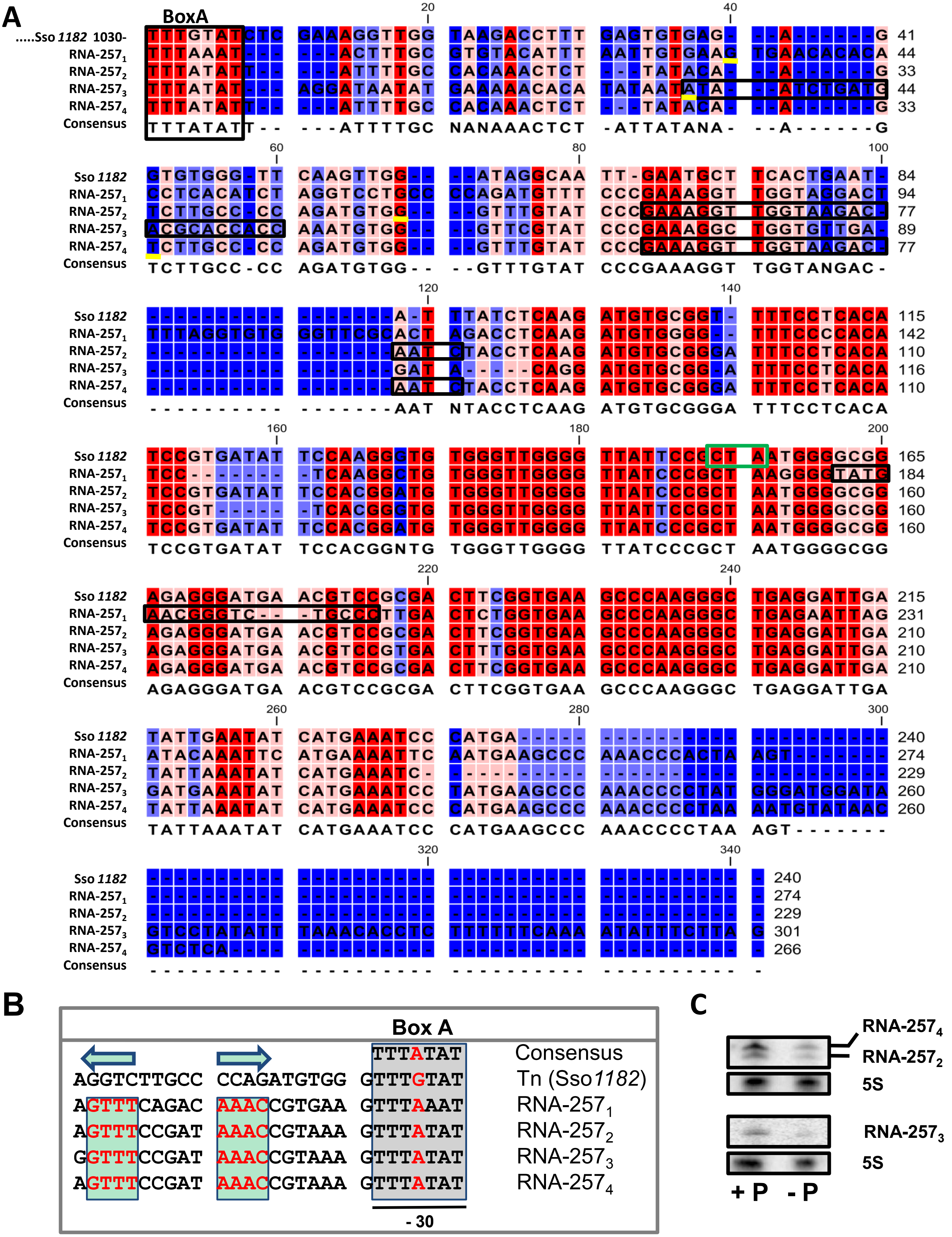

A
BoxA
.....Sso 1182 1030-
RNA-257₁
RNA-257₂
RNA-257₃
RNA-257₄
Consensus
B
Box A
TTTATAT Consensus
AGGTCTTGCC CCAGATGTGG GTTTGTAT Tn (Sso1182)
AGTTTCAGAC AAACCGTGAA GTTTAAAT RNA-257₁
AGTTTCCGAT AAACCGTAAA GTTTATAT RNA-257₂
GGTTTCCGAT AAACCGTAAA GTTTATAT RNA-257₃
AGTTTCCGAT AAACCGTAAA GTTTATAT RNA-257₄
- 30
C
RNA-257₄
RNA-257₂
5S
RNA-257₃
5S
+ P
- P

D

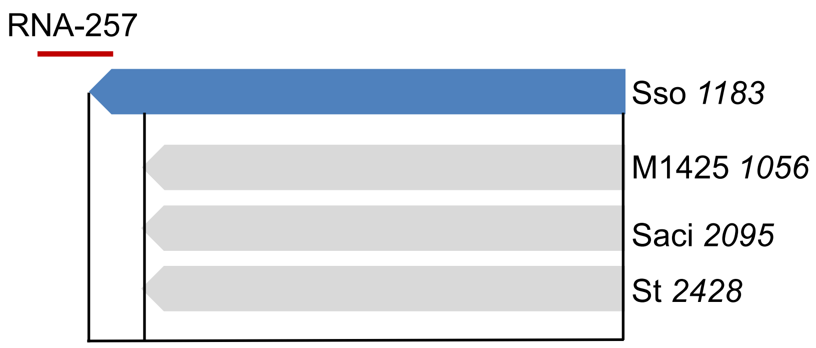

Fig S1 (A) Alignment of the 3 end of Sso ORF *1182* (from bp 1030 to bp 1269), RNA-257_1 and the 3 paralog RNAs, RNA-257_{2-4} (RNA-107, RNA-91 and RNA-20). The RNA-257_{1-4} paralogs differ in length but posses a highly conserved core (red) of approx. 100 nt (70-90 % identity) and show an overall sequence identity of >50 %. The alignment was performed using CLC sequence viewer software. The Box A promoter region is highlighted with a black box. The 5 end of RNA-257_1 (was mapped by a circularization approach; not shown.) and that of RNA-257_{2-4} [12] are underlined in yellow. The green box depicts the stop codon of Sso ORF *1183* encoded on the opposite DNA strand. The black box denotes the binding site for the RNA-257_1, RNA-257_2, RNA-257_3 and RNA-257_4 specific probes used for Northern-blot analyses. **(B)** Promoter region of RNA-257_{1-4} paralogs and the corresponding sequence in Sso ORF *1182*. Note that the sub-sequence in Sso ORF *1182* deviates from the Box A consensus sequence by the presence of a G. The inverted repeat located upstream of the Box A promotor region (approx. 30 bp) is highlighted in green. **C)** Determination of the RNA-257_2, RNA-257_3 and RNA-257_4 levels by Northern-blot analyses using total RNA of *Sso* grown in either full medium (+P) or under phosphate limiting conditions (-P). 5S rRNA was used as loading control. **(D)** Analysis of Sso *1183* orthologous genes in 3 different Sulfolobales; *S. islandicus* (M1425 *1056*); *S. acidocaldarius* (Saci *2095*); *S. tokodaii* (St *2428*). The last 90 nucleotides of Sso *ORF 1183* share homology only with the transposase sequence (3´ end of ORF *1182)* and not with the putative phosphate transporter genes of M1425 (*1056*); Saci (*2095)* or St (*2428)*, i.e. the base-pairing region (bar) for RNA-257_{1-4} with the 3´end and the 3´UTR of *1183* is only present in *Sso*.

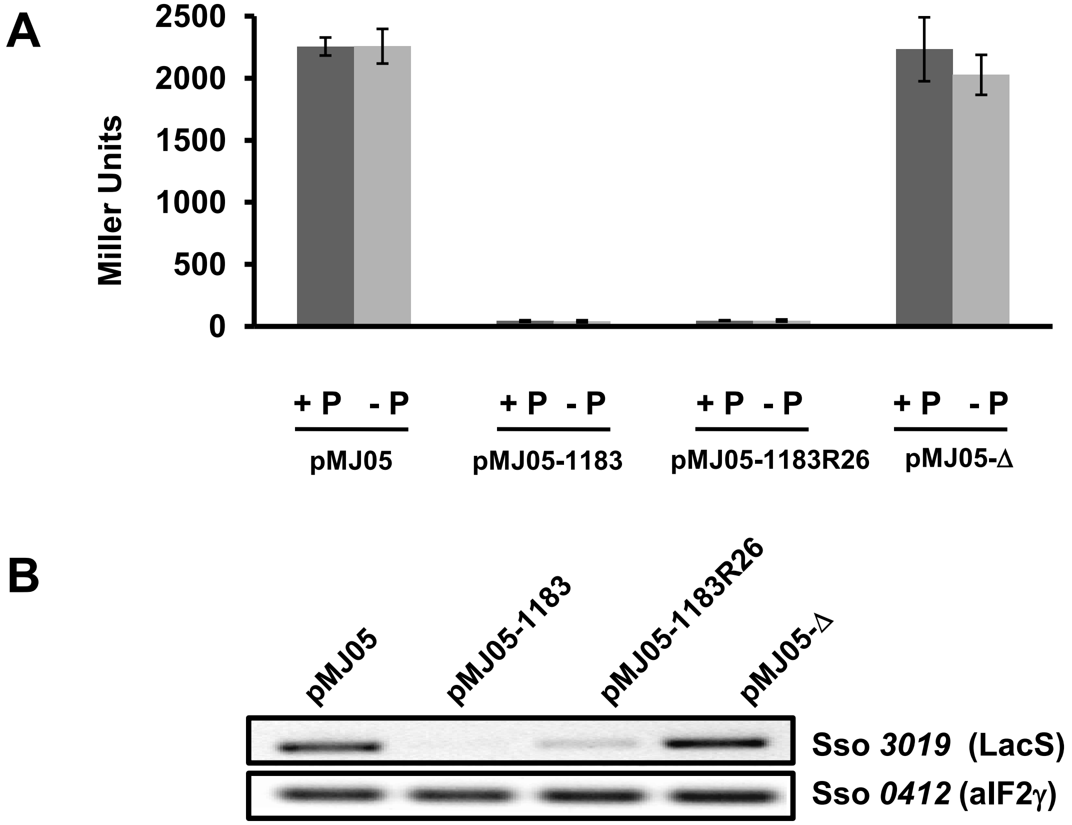

Fig S2 (A) β-Galactosidase activity determined in crude cell extracts of strains Sso PH1-16(pMJ05), Sso PH1-16(pMJ05-1183), Sso PH1-16(pMJ05-1183R26) and Sso PH1-16(pMJ05-Δ) grown in Brock´s medium (+P) and under phosphate limiting conditions (-P). The error bars represent standard deviations. **B)** RT-PCR performed with 10 ng cDNA obtained from strains PH1-16(pMJ05), PH1-16(pMJ05-1183), PH1-16(pMJ05-1183R26) and PH1-16(pMJ05-Δ) with *lacS* or Sso *0412* (house-keeping endogenous control) specific primers. The experiment was performed in triplicate. The result of one representative experiment is shown.

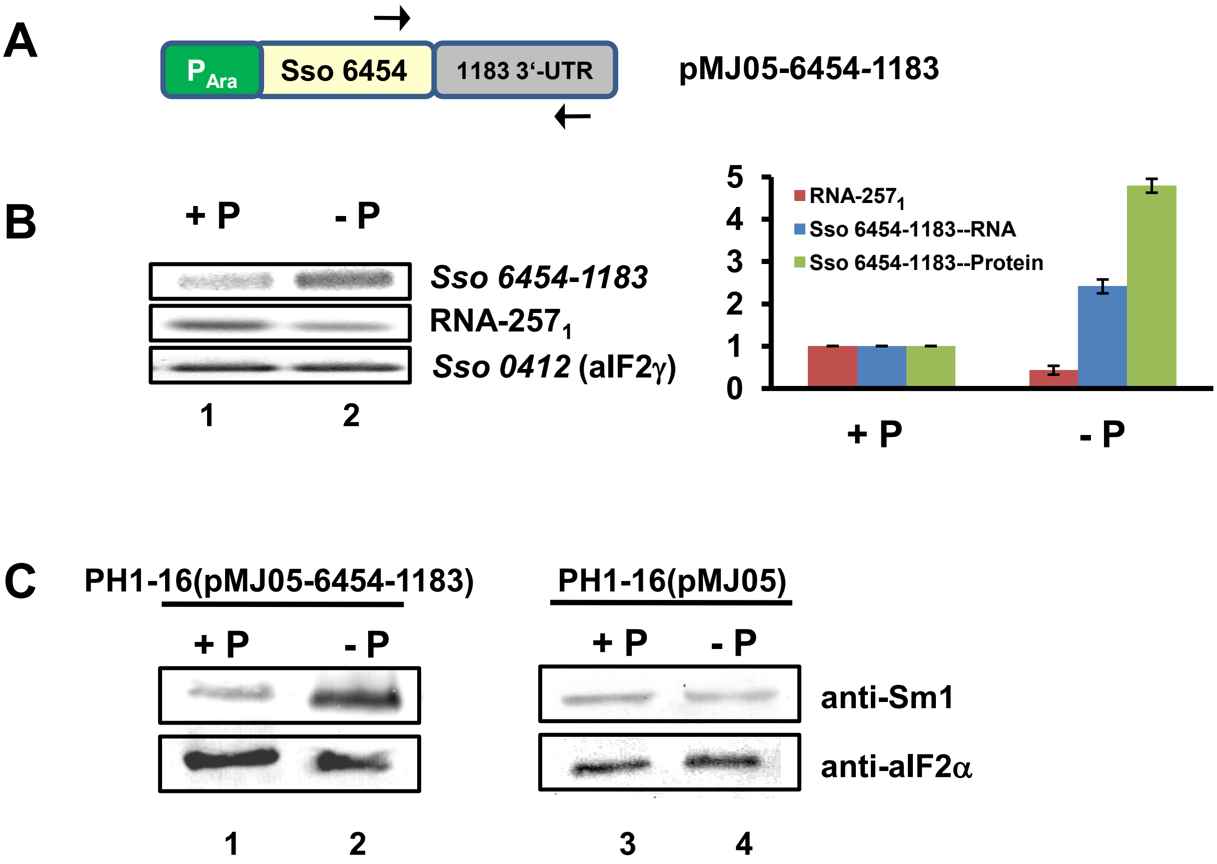

Fig S3 (A) Schematic depiction of the Sso *6454*-3´UTR-*1183* gene fusion. The arrows depict the binding positions of the oligonucleotides used for RT-PCR. **(B)** RT-PCR with cDNA derived from strain PH1-16(pMJ05-6454-1183) grown in full medium (+P; lane 1) and under phosphate limiting conditions (-P; lane 2), respectively, using oligonucleotides specific for the Sso *6454-1183* fusion, RNA-257_1 and Sso *0412* (house-keeping endogenous control). The experiment was performed in triplicate. The result of one representative experiment is shown. Right panel: Graphic representation of the levels of the RNA-257_1 transcript (see B; red), the Sso *6454-1183* transcript (see B; blue) and of the Sso 6454-1183 protein (Sm protein; see C; green) from three independent experiments using Image J software. The values for "+P" was set to 1; the error bars represent standard deviations. **(C)** Sso strain PH1-16(pMJ05-6454-1183) was grown in full medium (+P; lane 1) and under phosphate limiting conditions (-P; lane 2), respectively. Lanes 3 and 4, Sso strain PH1-16(pMJ05) (control) was grown in full medium (+P; lane 3) and under phosphate limiting conditions (-P; lane 4), respectively. The quantitative western-blot analyses were performed with anti-Sm1 antibodies and anti-aIF2α antibodies (internal control) following standard procedures.

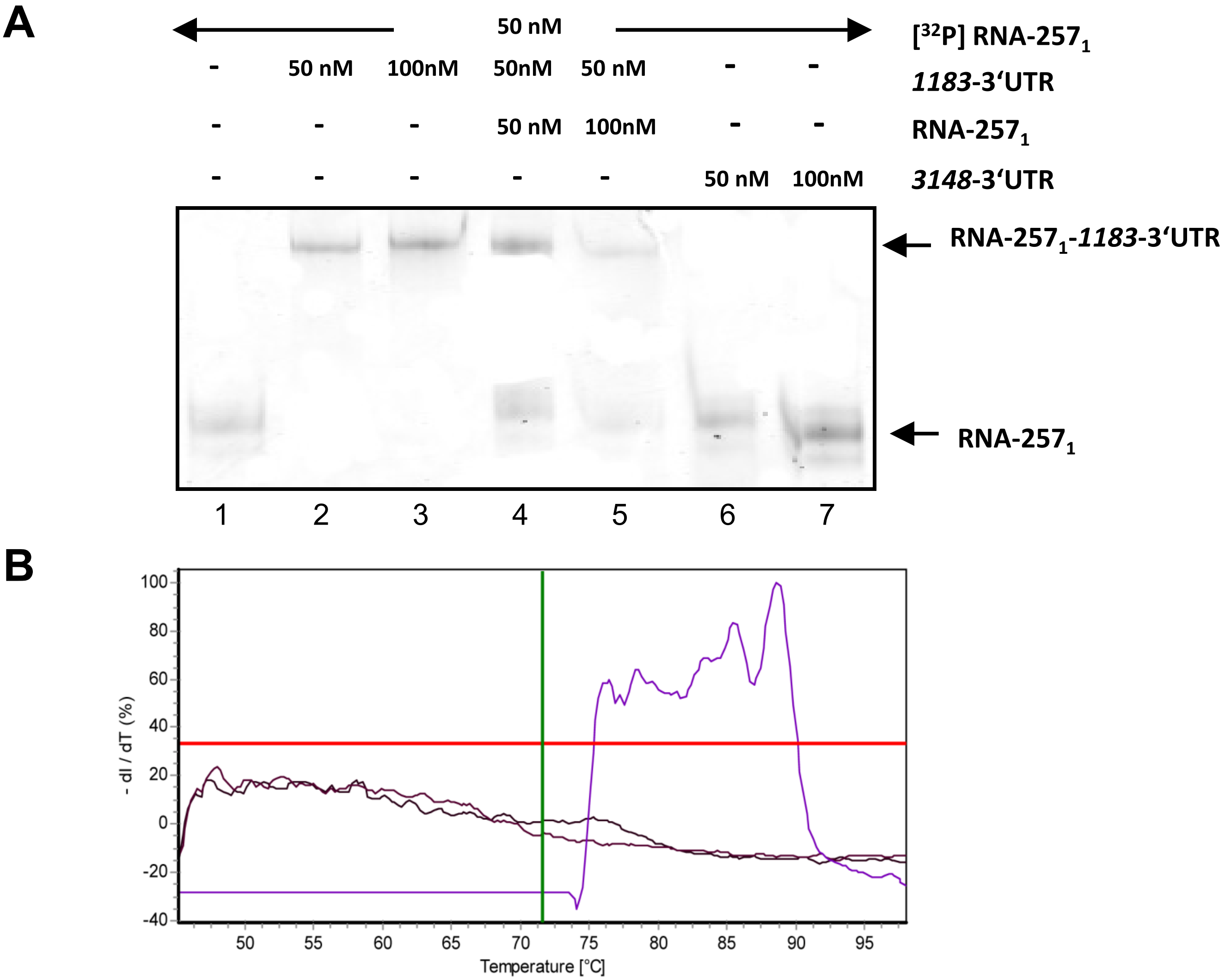

Fig S4 (A) Duplex formation of RNA-257$_1$ with the 3' end and 3 UTR of ORF *1183* (*1183*-3 UTR) determined by electrophoretic mobility shift assays. The [^{32}P] labelled and unlabelled RNA-257$_1$ as well as the ORF *1183*-3`UTR and the 3'UTR of ORF *3148* (negative control) were transcribed *in vitro* and gel-purified. The radiolabeled RNA-257$_1$ was incubated at 75 C for 5 min alone (lane 1), together with the *1183*-3'UTR (lanes 2-5) in the absence (lanes 2-3) or presence (lanes 4-5) of unlabeled RNA-257$_{1,}$ and together with *3148*-3'UTR (lanes 6-7), respectively. The RNAs were then subjected to native gel electrophoreses. The signals for RNA-257$_1$ and the RNA-257$_1$-*1183*-3'UTR duplex are indicated at the right by arrows. **(B)** The melting curve was established by using an Eppendorf mastercycler and 5 x qPCR mix (Medibena). 30 pmol *in vitro* transcribed RNA-257$_1$ (purple graph below red threshold), *1183*-3 UTR alone (black graph) or 30 pmol RNA-257$_1$ together with 30 pmol of *1183*-3 UTR (purple graph) were denatured for 5 min at 95 C. The melting curve analysis was performed by raising the temperature from 45°C to 98°C within 30 min, and by continuously measuring the fluorescence.
